# Disadvantaged children at greater relative risk of thinness (as well as obesity): a secondary data analysis of the England National Child Measurement Programme and the UK Millennium Cohort Study

**DOI:** 10.1186/s12939-015-0187-6

**Published:** 2015-08-05

**Authors:** Anna Pearce, Emeline Rougeaux, Catherine Law

**Affiliations:** Population Policy and Practice, UCL Institute of Child Health, 30 Guilford Street, London, WC1N 1EH UK

**Keywords:** Childhood thinness, Socio-economic inequalities, Early life characteristics

## Abstract

**Introduction:**

Young children living in more disadvantaged socio-economic circumstances (SECs) are at an increased risk of overweight and obesity. However, there is scant research examining the prevalence and social distribution of thinness in early childhood, despite potential negative consequences for health and development across the life-course.

**Methods:**

We examined the social gradient in thinness (and overweight and obesity for comparison) for 2,620,422 four-to-five year olds attending state maintained primary schools from 2007/8 to 2011/12, in the England National Child Measurement Programme (NCMP), and 16,715 children from the UK Millennium Cohort Study (MCS), born in 2000–2002, and measured at ages of three, five and seven. Children were classified as being thin, healthy weight (and, for completeness, overweight or obese) using international age and sex adjusted cut-offs for body mass index (BMI). Prevalences (and 95 % confidence intervals (CIs)) were estimated, overall, and according to SECs: area deprivation (NCMP, MCS); household income, and maternal social class and education (MCS only). Relative Risk Ratios (RRRs) and CIs for thinness, overweight and obesity were estimated in multinomial models by SECs (baseline healthy weight). In the MCS, standard errors were estimated using clustered sandwich estimators to account for repeated measures, and, for thinness, RRRs by SECs were also estimated adjusting for a range of early life characteristics.

**Results:**

In 2007/8 to 2011/12, 5.20 % of four-to-five year old girls (*n* = 66,584) and 5.88 % of boys (78,934) in the NCMP were thin. In the MCS, the prevalence of thinness was 4.59 % (693) at three, 4.21 % (702) at five, and 5.84 % (804) at seven years. In both studies, and for all measures of SECs, children from the most disadvantaged groups were more likely to be thin than those from the most advantaged groups. For example, MCS children whose mothers had no educational qualifications were fifty percent more likely to be thin (RRR 1.5 (CI: 1.24, 1.8)) than those whose mothers had a degree. These patterns were attenuated but remained after adjusting for early life characteristics.

**Conclusions:**

Children from more disadvantaged backgrounds are at elevated relative risk of thinness as well as obesity. Researchers and policymakers should consider environmental influences on thinness in addition to overweight and obesity.

**Electronic supplementary material:**

The online version of this article (doi:10.1186/s12939-015-0187-6) contains supplementary material, which is available to authorized users.

## Introduction

Food poverty (the inability to afford or access a healthy diet) is rising across the UK, as prices have increased and real incomes fallen. These trends have been linked to shifts in household expenditure away from healthier foods, and dramatic rises in referrals to food banks [1] (non-profit charitable organisations which provide emergency food packages to individuals in need). Families with young children are particularly susceptible to these trends [2–4]. However the burden of childhood thinness (equivalent of <18.5 body mass index [BMI] in adults, adjusting for age and sex [5]), has received little attention in the UK, despite potentially serious consequences for health, development and mortality across the life-course [6–8]. Regional studies in the UK [9, 10], and a nationally representative study in Scotland [11], have estimated the burden of childhood thinness to be around 7–8 %.

Studies in low-income countries have shown that low socio-economic status is associated with a greater risk of adult underweight [12], and that underweight and obesity coexist within poorer populations and families [13, 14]. A small number of studies in high income countries have examined whether thinness (as defined above [5]) in early childhood is socially patterned [11, 15, 16]; however these studies often employed small, non-representative samples, and findings have been mixed. Just two nationally representative studies have examined low BMI in early childhood in the UK, although both employed distribution based definitions of low BMI (<2^nd^ centile on UK90 BMI growth reference). The first found that preschool children living in more deprived areas in Scotland were more likely to have low BMI than those living in more advantaged areas [17]. The second found in a series of reports [18] that children attending primary schools in more deprived areas of England were more likely to have a low BMI than those attending school in the most advantaged areas. To our knowledge, there are no national estimates of the burden of thinness in early childhood in the UK, or studies examining inequalities according to individual level socio-economic circumstances, which use a child equivalent of the World Health Organisation (WHO) adult definition of underweight (BMI < 18.5).

There are a number of early life factors, such as birthweight, smoking in pregnancy, and breastfeeding, which are socially distributed and associated with growth in childhood. The Scotland-wide study in preschool children found that variations in low BMI according to area deprivation were only partially attenuated after adjustment for birthweight [17], although to our knowledge no study has accounted for other early life characteristics, possibly due to lack of data or statistical power.

The aim of this study was to examine the prevalence and social distribution of childhood thinness, as compared to overweight and obesity, using international age and sex specific cut-offs, and data from two contemporary and nationally representative datasets: the England National Child Measurement Programme (NCMP) and the UK Millennium Cohort Study (MCS). BMI status in childhood may be the result of early life exposures (such as maternal BMI, birthweight and infant feeding), more recent conditions (e.g. diet), or both. A secondary aim was therefore to examine the role of early life characteristics in explaining any socio-economic inequality in thinness (in the MCS).

## Methods

### Defining thinness

In 2007 Cole et al. devised age and sex specific BMI cut-offs for thinness, to supplement the International Obesity Task Force (IOTF) cut-offs for defining overweight and obesity in children. These cut-offs provide the first internationally validated classification of thinness in children over the age of 5 years, which unlike earlier distribution based definitions of low childhood BMI (e.g. < 2^nd^ centile on the UK90 BMI growth references), approximate the WHO definitions of adult underweight (BMI <18.5). These cut-offs allow for thinness to be further divided into mild (BMI 17.00 to <18.5), moderate (BMI 16.00 to <17.00) and severe (<16.00) thinness [5]. For completeness, we also present data on overweight and obesity, defined using IOTF cut-offs.

### The England National Child Measurement Programme (NCMP)

#### Participants

The England NCMP has collated the heights and weights of all 4–5 years (and 10–11 year old children) who attend state maintained primary schools (attended by approximately 95 % of 4 year old children) since 2006/7, with the aim of assessing national rates of childhood overweight and obesity. Local Authorities are responsible for overseeing data collection, and while participation is not mandatory, children or their parents must opt-out if they do not want to be included. In 2006/7, the heights and weights of 83 % of all 4–5 year old children enrolled at a state school were collected. The response rate has since improved, from 89 % in 2007/8 to 94 % in 2011/12 [18]. We analysed data for 2,620,422 4–5 year old children for the period 2007/8 to 2011/12 (we omitted data from the first year of collection due to low response and the high proportion of missing data on for child’s residential area deprivation [42 %]). NCMP data are provided by the Health and Social Care Information Centre (HSCIC), and these data were downloaded from the UK Data Service, University of Essex and University of Manchester, between August and October 2013.

#### Measures

Children were measured by trained staff (normally healthcare assistants) at school, without shoes and in light indoor clothing. Heights were measured, using stand-on height measures, to the nearest 0.1 cm. Weights were recorded with calibrated Class III weighing scales, to the nearest 0.1 kg [19]. Children were classified as moderately/severely thin, mildly thin (excluding moderate/severe), healthy, overweight or obese according to the international age and sex specific cut-offs for BMI (using the British 1990 growth reference) [5].

Area deprivation of the child’s residence, measured at the Super Output Area (SOA) [20] using the 2010 Index of Multiple Deprivation (IMD), was available in national deciles.

#### Analysis

Prevalence and 95 % confidence intervals (CIs) of mild and moderate/severe thinness (as well as healthy weight, overweight and obesity) were estimated for each year, according to the child’s gender, and by area deprivation. We estimated relative risk ratios (RRRs) and CIs for being mildly thin, moderately/severely thin, overweight and obese (baseline healthy weight) using multinomial logistic regression, adjusting for year. Child’s ethnicity was not accessible in the NCMP dataset. The association between area deprivation and childhood thinness varied very little by year, so analyses are presented for all years combined.

### The UK Millennium Cohort Study (MCS)

#### Participants

The MCS is a longitudinal study of children born in the UK between September 2000 and January 2002. A disproportionately stratified clustered sampling design was used to over-represent children living in Wales, Scotland and Northern Ireland, disadvantaged areas, and in the case of England, areas with high proportions of ethnic minority groups [21]. Families were selected through Child Benefit Records, and initially contacted via opt-out letters from the Department for Work and Pensions. The first study contact with the cohort child was carried out at around age 9 months. Interviews were carried out by trained interviewers in the home with the main respondent (usually the mother). Information was collected from 18,818 infants (91 % of the 20,646 in the target sample), of which 18,296 were singletons. For subsequent sweeps, participants were followed up by letter, telephone (when possible), and a visit. An additional 685 children entered into the study at the second sweep, when the children were age three, in order to boost the sample. We analyse data collected in infancy and at ages three (carried out in 2003–5, *n* = 15,381), five (2006–7, *n* = 15,041) and seven (2008, *n* = 13,681) years of age. Our analysis was limited to singleton children who had BMI data in at least one of the relevant sweeps (*n* = 16,715, 88 % of all singletons). Data were downloaded from the UK Data Service, University of Essex and University of Manchester, in April 2014.

#### Measures

BMI was available at ages three, five and seven, from height and weight data collected by trained interviewers in the home. Children were weighted with Tanita HD-305 scales (Tanita UK Ltd., Middlesex, UK), without shoes or outdoor clothing; weights were recorded in kilograms to one decimal place. Heights were measured with the Leicester Height Measure Stadiometer (Seca Ltd., Birmingham, UK) and recorded to the nearest millimetre [22]. Cole’s international age and sex specific cut-offs for BMI were used to classify children as thin (mild, moderate and severe combined, due to limited numbers), healthy, overweight or obese [5].

We examined a number of individual level measures representing the child’s socio-economic circumstances (SECs). Mother’s social class (National Statistical Socio-economic Classification [NS-SEC]) in her current or last known employment was captured when the children were aged 9 months and grouped as follows: managerial and professional, intermediate, routine and manual, and never worked/long-term (L/T) unemployed. We also examined two time varying measures of SECs (based at status at age 3, 5 and 7 years): maternal highest academic qualification, classified as degree and above, diploma, A-Levels, GCSE grades A*-C, GCSE grades D-G, other qualifications, and no qualifications; and equivalised household income divided into quintiles (missing income data were multiply imputed by the data owners). Finally, IMD was used to assess area deprivation, in order to be able to compare findings to the NCMP. IMD was based on the SOA of the child’s postcode at age three, five and seven, divided into national quintiles (in England only).

We explored a number of early life characteristics that were reported at 9 months and are known to be socially distributed and might be associated with weight status: maternal pre-pregnancy BMI (derived from self-reported heights and weights; categorised with WHO cut-offs as underweight, healthy, overweight, obese, morbidly obese); mother’s age at birth of the cohort child (years); smoking in pregnancy (never smoked, smoked but stopped at pregnancy, smoked 1–5, 6–10, and 11+ cigarettes a day during pregnancy [based on the distribution of the data]); alcohol consumption in pregnancy (using groups recommended by Kelly et al. [23], which relate to the National Alcohol Strategy criteria: never, light [up to 2 units per week or on an occasion], moderate [3/4 units per week or on an occasion], heavy/binge [5 or more units per week or on an occasion] drinking; birthweight (low [<2.5 kg], normal [> = 2.5 kg/ <=4.5 kg] or high [>4.5 kg]; gestational age (weeks); parity; breastfeeding duration (based on government recommendations at that time [24]: never, for less than 4 months, for more than 4 months).

We also examined ethnicity of the child, categorised as: White or White British; Mixed ethnicity; Black or Black British; Indian; Pakistani or Bangladeshi; and ‘Other’ ethnicities (including Chinese).

#### Analysis

We estimated prevalence (and 95 % CIs) of thinness, healthy weight, overweight and obesity, using survey and response weights at each age, overall and according to SECs (social class at 9 months, and maternal education, income and area deprivation at concurrent sweep). We estimated the overall prevalence of weight status (across all ages) using *xt* commands (for analysing panel data) in Stata. Data for all sweeps were then pooled (to maximise power) and RRRs (and 95 % CIs) were estimated in multinomial logistic regression models for thinness, (and overweight and obesity), baseline healthy weight. We accounted for the repeated measures within children using a clustered sandwich estimator. Following this, we estimated associations for thinness, before and after adjustment for early life factors. Of the 16,715 children who were included in the main analysis, 669 were excluded because they did not take part at MCS1, and a further 30 because the main respondents were not a natural mother at MCS1. Of the remaining 16,016, 1501 (9 %) were missing one or more of the covariates. These children were more likely to be thin or obese and less likely to be overweight or of healthy weight than children who were not missing covariates. Gestational age was entered as a continuous variable into the regression models but is presented as a categorical measure in the descriptive statistics (Additional file 1). Missing data for each variable are listed under the relevant tables.

Inequality in thinness varied little by gender; therefore MCS analyses were carried out for both sexes combined to maximise power. It was not possible to adjust for repeated measures in the pooled models as well as sample design and attrition; however all analyses were repeated for each sweep individually, adjusting for sample design and attrition (and patterns remained similar). Children from Indian, Pakistani, Bangladeshi and ‘Other’ ethnicities had a considerably higher prevalence of thinness than White children; they were more likely to come from disadvantaged backgrounds, and the social gradient of thinness appeared to be different in these groups (although did not reach significance). We therefore present additional analyses for thinness, limited to children from White, Black or mixed ethnicity backgrounds, in order to remove the potential confounding of South Asian (Indian, Pakistani and Bangladeshi) and ‘Other’ ethnicities. A further sensitivity analysis was carried out excluding children of mixed ethnicities that included Asian; the results were unchanged and so are not presented in the paper. As seen in previous studies in the MCS [25] and elsewhere [18], children from some South Asian backgrounds were also more likely to be obese than White children.

The international age and sex specific BMI cut-offs were assigned using the LMS Growth package in Excel [5]. All analyses were carried out in Stata 13. Response and attrition weights were applied to some of the MCS analyses (where indicated), using *sv*y commands in Stata.

The present analyses did not require ethics approval.

## Results

### NCMP

Across the period 2007/8 to 2011/12, 5.2 % (95 % CI: 5.16, 5.24, *n* = 66,584) of 4–5 year old girls, and 5.88 % (5.85, 5.93, *n* = 78,934) of boys living in England were thin (of which around one fifth were moderately/severely thin). This amounted to almost 160,000 children over the 5-year period (Table 1). The prevalence of thinness was relatively stable across the period, although small declines were observed between 2007/8 and 2009/10. Boys were more likely to be mildly thin than girls, although the prevalence of moderate/severe thinness was similar for both sexes.

**Table 1:** Percentage (95 % CI); number (n) who were thin, healthy weight, overweight or obese (NCMP, age 4–5 years)

	2007/8	2008/9	2009/10	2010/11	2011/12	2007/8- 2011/12
Girls						
Moderately/severely thin	1.14 (1.10, 1.19); 2 670	0.90 (0.86, 0.93); 2 215	0.81 (0.77, 0.84); 2 083	0.87 (0.83, 0.90); 2 295	0.78 (0.75, 0.81); 2 162	0.89 (0.88, 0.91); 11 425
Mildly thin	4.71 (4.62, 4.79); 10 980	4.29 (4.21, 4.37); 10 623	4.05 (3.98, 4.13); 10 441	4.34 (4.26, 4.42); 11 499	4.20 (4.12, 4.27); 11 616	4.31 (4.27, 4.34); 55 159
Healthy weight	73.07 (72.89, 73.25); 170 399	73.36 (73.19, 73.35); 181 545	73.35 (73.18, 73.52); 188 914	73.53 (73.36, 73.70); 194 771	73.43 (73.26, 73.59); 203 209	73.36 (73.28, 73.34); 938 838
Overweight	15.99 (15.84, 16.14); 37 283	16.38 (16.24, 16.53); 40 539	16.50 (16.36, 16.65); 42 503	16.25 (16.11, 16.39); 43 045	16.40 (16.26, 16.54); 45 387	16.31 (16.25, 16.38); 208 757
Obese	5.09 (5.00, 5.18); 11 860	5.07 (4.98, 5.16); 12 547	5.29 (5.20, 5.37); 13 614	5.01 (4.93, 5.09); 13 274	5.20 (4.93, 5.09); 14 379	5.13 (5.09, 5.17); 65 674
Total n	233 192	247 469	257 555	264 884	276 753	1 279 853
Boys						
Moderately/severely thin	1.10 (1.06, 1.14); 2 685	0.88 (0.85, 0.92); 2 294	0.82 (0.79, 0.85); 2 211	0.86 (0.82, 0.89); 2 370	0.79 (0.76, 0.82); 2 292	0.88 (0.87, 0.90); 11 852
Mildly thin	5.38 (5.29, 5.47); 13 168	4.97 (4.89, 5.06); 12 893	4.81 (4.73, 4.89); 12 957	4.97 (4.89, 5.05); 13 772	4.93 (4.85, 5.01); 14 292	5.00 (4.97, 5.04); 67 082
Healthy weight	77.46 (77.30, 77.63); 189 581	78.14 (77.98, 78.30); 202 651	78.19 (78.03, 78.34); 210 800	78.34 (78.19, 78.49); 217 124	78.67 (78.53, 78.82); 227 936	78.18 (78.11, 78.25); 1 048 092
Overweight	12.20 (12.07, 12.33); 29 856	12.34 (12.22, 12.47); 32 014	12.33 (12.21, 12.46); 33 251	12.13 (12.01, 12.25); 33 627	11.96 (11.85, 12.08); 34 662	12.19 (12.13, 12.24); 163 410
Obese	3.86 (3.78, 3.94); 9 444	3.66 (3.59, 3.73); 9 494	3.85 (3.78, 3.93); 10 389	3.70 (3.63, 3.77); 10 267	3.64 (3.57, 3.71); 10 539	3.74 (3.71, 3.77); 50 133
Total n	244 734	259 346	269 608	277 160	289 721	1 340 569

Table 2 shows RRRs for being moderately/severely thin, mildly thin, overweight or obese (baseline healthy weight) for all years combined, by area deprivation and gender. Children living in more deprived areas were at a greater relative risk of thinness (and overweight and obesity) compared to children living in the least deprived areas. The degree of relative inequality was greatest for obesity, followed by moderate/severe thinness. The relative risk of thinness increased with area deprivation across the entire socio-economic gradient (similar to patterns by overweight and obesity), whereas the elevated relative risk of mild thinness were only apparent in the most disadvantaged half the distribution.

**Table 2:** Percentage (n) and RRRs (95 % CIs) for weight status by area deprivation (baseline healthy weight) (NCMP, age 4–5 years)

	Moderately/severely thin		Mildly thin		Overweight		Obese	
IMD decile	% (n)	RRR (95 % CI)	% (n)	RRR (95 % CI)	% (n)	RRR (95 % CI)	% (n)	RRR (95 % CI)
Girls								
Most advantaged	0.71 (855)	-	4.14 (4 998)	-	14.45 (17 426)	-	2.95 (3 563)	-
2	0.76 (868)	1.08 (0.98, 1.19)	4.01 (4 606)	0.98 (0.94, 1.02)	15.09 (17 334)	1.06 (1.04, 1.08)	3.36 (3 980)	1.19 (1.14, 1.25)
3	0.74 (812)	1.07 (0.97, 1.17)	4.01 (4 426)	0.99 (0.95, 1.03)	15.46 (17 055)	1.09 (1.07, 1.12)	3.84 (4 236)	1.33 (1.27, 1.39)
4	0.75 (805)	1.10 (1.00, 1.21)	4.02 (4 315)	1.00 (0.96, 1.05)	15.97 (17 138)	1.14 (1.11, 1.17)	4.04 (4 334)	1.41 (1.35, 1.48)
5	0.79 (876)	1.17 (1.07, 1.29)	4.0 (4 408)	1.01 (0.97, 1.05)	16.12 (17 761)	1.16 (1.14, 1.19)	4.53 (4 990)	1.60 (1.53, 1.67)
6	0.86 (991)	1.28 (1.17, 1.41)	4.10 (4 739)	1.05 (1.00, 1.09)	16.53 (19 121)	1.21 (1.19, 1.24)	5.00 (5 784)	1.79 (1.72, 1.87)
7	0.89 (1 089)	1.37 (1.25, 1.50)	4.41 (5 374)	1.15 (1.11, 1.20)	17.06 (20 768)	1.28 (1.25, 1.31)	5.60 (6 816)	2.05 (1.96, 2.13)
8	1.02 (1 370)	1.57 (1.44, 1.71)	4.5 (6 042)	1.18 (1.14, 1.23)	16.77 (22 529)	1.27 (1.25, 1.30)	6.11 (18 210)	2.25 (2.16, 2.34)
9	1.06 (1 605)	1.67 (1.53, 1.81)	4.8 (7 254)	1.28 (1.24, 1.33)	17.25 (26 091)	1.34 (1.31, 1.37)	6.77 (10 238)	2.54 (2.44, 2.64)
Least advantaged	1.14 (2 009)	1.80 (1.66, 1.95)	4.73 (8 317)	1.27 (1.23, 1.32)	17.36 (30 561)	1.36 (1.34, 1.39)	7.18 (12 630)	2.71 (2.61, 2.82)
Total n	11 280		54 479		205 784		64 781	
Boys								
Most advantaged	0.65 (828)	-	4.88 (6 183)	-	9.97 (12 618)	-	2.03 (2 573)	-
2	0.68 (822)	1.05 (0.96, 1.16)	4.64 (5 608)	0.96 (0.93, 1.00)	10.71 (12 932)	1.08 (1.06, 1.11)	2.42 (2 920)	1.20 (1.14, 1.27)
3	0.67 (775)	1.05 (0.95, 1.16)	4.59 (5 295)	0.96 (0.92, 1.00)	11.22 (12 958)	1.14 (1.11, 1.17)	2.63 (3 033)	1.32 (1.25, 1.39)
4	0.75 (839)	1.18 (1.07, 1.30)	4.77 (5 352)	1.01 (0.97, 1.05)	11.61 (13 036)	1.20 (1.17, 1.23)	2.89 (3 243)	1.47 (1.39, 1.55)
5	0.78 (911)	1.25 (1.14, 1.38)	4.77 (5 544)	1.02 (0.98, 1.06)	11.93 (13 863)	1.25 (1.22, 1.28)	3.23 (3 748)	1.65 (1.57, 1.74)
6	0.84 (1 026)	1.37 (1.25, 1.50)	5.01 (6 088)	1.08 (1.05, 1.12)	12.30 (14 947)	1.31 (1.28, 1.34)	3.74 (4 539)	1.94 (1.85, 2.04)
7	0.91 (1 153)	1.49 (1.36, 1.63)	4.98 (6 328)	1.09 (1.05, 1.13)	12.82 (16 285)	1.38 (1.35, 1.41)	4.08 (5 188)	2.15 (2.05, 2.25)
8	1.03 (1 443)	1.71 (1.56, 1.86)	5.16 (7 242)	1.14 (1.10, 1.18)	13.02 (18 286)	1.43 (1.40, 1.47)	4.54 (6 384)	2.42 (2.31, 2.54)
9	1.11 (1 765)	1.87 (1.72, 2.04)	5.36 (8 504)	1.21 (1.17, 1.25)	13.33 (21 170)	1.49 (1.46,1.53)	5.08 (8 066)	2.75 (2.62, 2.87)
Least advantaged	1.16 (2 125)	1.97 (1.81, 2.13)	5.49 (10 062)	1.25 (1.21, 1.29)	13.63 (24 989)	1.54 (1.51,1.58)	5.33 (9 769)	2.91 (2.78, 3.04)
Total n	11 687		66 206		161 084		49 463	

Adjusted for year. Missing IMD data: 75 724 (1.48 %) overall, ranging from 4.87 % in 2007/8 to 0.30 % in 2010/11

### MCS

The average prevalence of thinness across all time points in the MCS was 5.22 % (the majority were mildy thin (4.21 %) and 0.97 % were moderately-severely thin). 16.22 %were overweight and 5.68 % were obese. Below are weighted proportions for each individual sweeps (accounting for the sample design and attrition). These indicate little variation in weight status between ages three and five, and a small increase in thinness by age seven:

- Three years: 4.59 % (95 % CI: 4.20, 5.02, *n* = 693) of children were thin (3.61 % mild; 0.87 moderate-severe); 17.88 % (17.18, 18.59; *n* = 2530) overweight, and 5.01 % (4.58, 5.47; *n* = 735) obese
- Five years: 4.21 % (3.79, 4.67; *n* = 702) of children were thin (3.52 % mild; 0.69 moderate-severe) 15.42 % (14.74, 16.13, *n* = 2345) overweight, and 5.55 % (5.10, 6.03; *n* = 858) obese
- Seven years: 5.84 % (5.33, 6.40; *n* = 804) of children were thin (3.61 % mild, 1.01 % moderate-severe); 14.49 % (13.86, 15.15; *n* = 1959) overweight, and 5.64 % (5.20, 6.13; *n* = 800) obese

When South Asian/’Other’ ethnicities were excluded the prevalence of thinness was 3.65 % (3.31, 4.03; *n* = 430) at age three, 3.50 % (3.11, 3.94; *n* = 457) at five and 4.91 % (4.47, 5.39; *n* = 553) at seven.

An elevated relative risk of thinness was only seen in the most disadvantaged groups (Table 3). As with NCMP, inequality in obesity increased with disadvantage across the socio-economic gradient; relative inequalities in thinness were smaller than for obesity, but larger than overweight. Inequalities in thinness were attenuated when analyses excluded children from South Asian or ‘Other’ ethnic categories, although for some measures of SECs, the elevated relative risk of thinness in the most disadvantaged groups remained (Table 3, final column).

**Table 3:** Percentage (n) and RRRs (95 % CIs) for weight status (baseline healthy weight) by socio-economic circumstances (MCS, age 3, 5, 7 years’ sweeps combined)

	All children						Excluding South Asian and ‘Other’ ethnic categories	
	Thin % (n)	RRR Thin	Overweight % (n)	RRR Overweight	Obese % (n)	RRR Obese	% (n) Thin	RRR Thin
Maternal education								
Degree	6.34 (406)	-	16.57 (1,190)	-	4.78 (301)	-	5.21 (308)	-
Diploma	5.29 (178)	0.83 (0.66, 1.03)	18.08 (703)	1.11 (0.98, 1.27)	6.82 (233)	1.46 (1.15, 1.84)	4.68 (148)	0.89 (0.7, 1.14)
A Level	5.93 (190)	0.94 (0.74, 1.18)	16.83 (610)	1.01 (0.89, 1.16)	5.43 (173)	1.15 (0.89, 1.45)	4.78 (139)	0.92 (0.71, 1.2)
GCSE A*-C	6.09 (633)	0.96 (0.81, 1.14)	19.19 (2,318)	1.19 (1.08, 1.31)	7.42 (783)	1.6 (1.34, 1.92)	4.89 (474)	0.94 (0.78, 1.14)
GCSE D-G	5.64 (179)	0.89 (0.71, 1.12)	19.31 (716)	1.2 (1.05, 1.36)	8.45 (276)	1.84 (1.47, 2.32)	4.69 (136)	0.90 (0.7, 1.17)
Other academic	11.33 (181)	1.88 (1.48, 2.39)	16.56 (281)	1.01 (0.85, 1.2)	9.35 (146)	2.04 (1.56, 2.69)	6.27 (66)	1.21 (0.87, 1.68)
None	9.09 (427)	1.5 (1.24, 1.8)	18.8 (989)	1.15 (1.03, 1.3)	10.03 (476)	2.24 (1.83, 2.73)	6.50 (228)	1.29 (1.03, 1.61)
Income quintiles (household equivalised)								
Top 20 %	5.86 (364)	-	16.4 (1,147)	-	5.00 (308)	-	5.36 (318)	-
2	5.40 (337)	0.92 (0.78,1.09)	19.25 (1,406)	1.22 (1.1,1.34)	6.57 (415)	1.34 (1.13,1.59)	4.73 (281)	0.88 (0.74,1.05)
3	5.86 (378)	1.00 (0.84,1.19)	18.74 (1,400)	1.18 (1.07,1.3)	7.07 (462)	1.45 (1.21,1.72)	4.58 (273)	0.85 (0.7,1.03)
4	7.42 (516)	1.3 (1.1,1.52)	17.79 (1,393)	1.1 (1,1.2)	8.25 (579)	1.71 (1.44,2.03)	5.11 (295)	0.96 (0.8,1.15)
Bottom 20 %	8.63 (595)	1.53 (1.3,1.8)	18.7 (1,449)	1.17 (1.06,1.29)	8.87 (613)	1.85 (1.56,2.2)	6.07 (331)	1.15 (0.96,1.39)
IMD quintiles (England only)								
Top 20 %	5.83 (215)	-	16.43 (683)	-	3.85 (139)	-	5.48 (193)	-
2	6.50 (227)	1.12 (0.88, 1.44)	16.02 (623)	0.97 (0.84, 1.12)	5.11 (176)	1.35 (1.01, 1.81)	5.72 (186)	1.05 (0.8, 1.34)
3	5.85 (222)	1.01 (0.79, 1.29)	18.15 (792)	1.12 (0.98, 1.29)	6.57 (251)	1.76 (1.34, 2.32)	4.34 (146)	0.79 (0.6, 1.03)
4	8.00 (347)	1.41 (1.13, 1.78)	17.97 (874)	1.11 (0.97, 1.27)	7.94 (344)	2.17 (1.67, 2.81)	5.60 (202)	1.03 (0.8, 1.34)
Bottom 20 %	10.32 (634)	1.88 (1.52, 2.31)	17.98 (1,208)	1.11 (0.98, 1.26)	9.76 (596)	2.72 (2.13, 3.48)	5.66 (233)	1.05 (0.82, 1.34)
Maternal social class (NS-SEC)								
I (Managerial & prof.)	5.54 (522)	-	17.88 (1,937)	-	5.77 (545)	-	4.90 (442)	-
II	6.17 (424)	1.12 (0.94, 1.33)	17.05 (1,326)	0.94 (0.86, 1.04)	6.21 (427)	1.08 (0.81, 1.57)	4.93 (315)	1.01 (0.83, 1.22)
III	6.16 (780)	1.12 (0.97, 1.31)	19.28 (2,835)	1.1 (1.01, 1.19)	8.13 (1,051)	1.45 (1.25, 1.67)	5.14 (592)	1.06 (0.9, 1.24)
L/T unemployed	13.19 (379)	2.6 (2.16, 3.12)	17.25 (520)	0.96 (0.84, 1.09)	10.41 (290)	1.9 (1.55, 2.33)	7.56 (114)	1.6 (1.22, 2.1)

Sample (total observations, children): Maternal education: 29136, 12786; income: 29064, 12783; Social class: 28426, 12415; IMD: 17856, 7750

Missing data: Education: 56 (all ages); Social class (9 m 596); Income: 3y (1689), 5y (1840), 7y (3008); IMD (England only): 3y (1), 5y (1), 7y (2), ethnicity: 104

A number of early life characteristics associated with disadvantage were also associated with higher risk of thinness: low birthweight, maternal pre-pregnancy underweight, and pre-term birth. On the other hand, thinness was also associated with older maternal age at birth and drinking in pregnancy, which tend to be more prevalent in advantaged groups (Additional file 1). We adjusted for these factors, in order to examine the extent to which they might explain or conceal any socio-economic inequality in thinness (Table 4); the degree of inequality remained similar, and in the majority of cases RRRs increased. These same patterns were seen when South Asian and ‘Other’ ethnicities were excluded, only the level of inequality was smaller.

**Table 4:** RRRs (95 % CIs) for thinness by SECs, before and after adjustment for pre- and post-natal covariates (MCS, age 3, 5 and 7 years’ sweeps combined), *N* = 14515

	Unadjusted	Adjusted^a^	Unadjusted (excluding South Asian and ‘Other’ ethnicities)	Adjusted^a^ (excluding South Asian and ‘Other’ ethnicities)
Maternal education				
Degree	-	-		-
Diploma	0.84 (0.66,1.06)	0.86 (0.68,1.09)	0.92 (0.71, 1.19)	0.95 (0.73,1.23)
A Level	0.9 (0.7,1.15)	0.94 (0.73,1.21)	0.91 (0.69, 1.2)	0.97 (0.74,1.29)
GCSE A*-C	0.91 (0.76,1.08)	1 (0.83,1.19)	0.93 (0.76, 1.13)	1 (0.82,1.23)
GCSE D-G	0.9 (0.7,1.15)	1.08 (0.83,1.39)	0.95 (0.73, 1.24)	1.11 (0.83,1.48)
Other academic	1.72 (1.31,2.27)	1.81 (1.37,2.41)	1.31 (0.92, 1.87)	1.47 (1.03,2.1)
None	1.46 (1.19,1.8)	1.68 (1.32,2.13)	1.31 (1.02, 1.67)	1.49 (1.12,1.99)
Income quintiles (household equivalised)				
Top 20 %	-	-		-
2	0.91 (0.76,1.08)	0.95 (0.79,1.13)	0.88 (0.73, 1.06)	0.93 (0.77,1.13)
3	0.97 (0.81,1.16)	1.07 (0.89,1.3)	0.86 (0.7, 1.05)	0.96 (0.78,1.18)
4	1.27 (1.07,1.5)	1.47 (1.22,1.77)	0.97 (0.8, 1.17)	1.13 (0.92,1.4)
Bottom 20 %	1.46 (1.22,1.74)	1.74 (1.43,2.12)	1.15 (0.94, 1.4)	1.35 (1.08,1.7)
IMD quintiles (England only)				
Top 20 %	-	-		-
2	1.15 (0.89,1.49)	1.17 (0.91,1.52)	1.05 (0.8, 1.39)	1.06 (0.8,1.39)
3	0.94 (0.72,1.23)	0.94 (0.72,1.23)	0.73 (0.54,0.97)	0.72 (0.54,0.97)
4	1.42 (1.12,1.81)	1.47 (1.14,1.88)	0.99 (0.75, 1.3)	0.99 (0.74,1.31)
Bottom 20 %	1.74 (1.38,2.18)	1.78 (1.4,2.27)	0.94 (0.72, 1.23)	0.96 (0.72,1.27)
Maternal social class (NS-SEC)				
I (Managerial & prof.)	-	-		-
II	1.12 (0.93,1.34)	1.16 (0.97,1.4)	0.99 (0.81, 1.2)	1.05 (0.86,1.29)
III	1.11 (0.95,1.3)	1.31 (1.11,1.56)	1.08 (0.92, 1.28)	1.27 (1.05,1.54)
L/T unemployed	2.56 (2.07,3.16)	2.78 (2.19,3.53)	1.64 (1.21, 2.21)	2 (1.43,2.79)

Sample, all children (total observations, children): Maternal education: 28805, 12613; income: 28719, 12603; Social class: 28671, 12547; IMD: 18066, 7819

Sample, excluding South Asian and ‘Other’ ethnicities (total observations, children): Maternal education: 26329, 11506; income: 26257, 11500; Social class: 26219, 11453; IMD: 15709, 6768

^a^Adjusting for mother’s pre-pregnancy BMI, maternal age at MCS birth, smoking in pregnancy, alcohol in pregnancy, birthweight, gestational age, breastfeeding

## Discussion

### Summary of findings

This paper aimed to examine the social gradient in childhood thinness, and the extent to which any differences might be explained by early life characteristics. In two large and nationally representative datasets, we found the prevalence of thinness in early childhood to be around 5–6 %. Similar prevalences have been shown in studies carried out in UK countries [17] and other high-income countries such as Australia [7, 16] and Sweden [26] , while levels appear to be higher in studies from lower income European countries [27, 28]. Socio-economic inequalities in thinness were observed in both datasets and with various measures of SECs; inequalities in thinness were smaller than obesity, but larger than overweight. When MCS analyses were limited to those not in South Asian and ‘Other’ ethnic groups (who were at higher risk of both thinness and disadvantage), inequalities in thinness were attenuated but remained. Adjustment for early life characteristics, such as birthweight and breastfeeding, did not alter these patterns.

### Comparison with other research

Obesity and thinness in low income countries have been shown to coexist within families and in poorer groups [13, 14], and this is thought to point towards similar causal mechanisms related to diet, physical activity and socio-demographic environment [13]. In high income countries, the socio-economic gradient in obesity is well established. A small number of studies have reported inequalities in childhood thinness, although many using small and non-representative samples. In Scotland in the late 1990s, thinness (<2^nd^ centile on the UK90 growth BMI references) was examined for 7 groups of area deprivation, and young children living in the two most deprived areas had higher odds of being thin than in the least deprived, with no indication of elevated odds for the groups in between [17]. In contrast, the North-East Scotland study of primary school aged children found no association between quintiles of school area deprivation and thinness in the 2000s [11]. Similarly, a study of primary school children in Australia [16] and another in Sweden [26] found no differences in thinness (according to area measures of deprivation in Australia, and education levels in Sweden), although in both cases socio-economic measures were broadly categorised as low, medium and high, which may have concealed differences between the most and least advantaged.

To our knowledge, ours is the first study to estimate the burden of thinness in England throughout early childhood, using the international age and sex standardised cut-offs. Descriptive statistics presented in the NCMP reports estimate that around 1 % of children have a BMI <2^nd^ centile on the British 1990 growth charts, and that levels are slightly higher than the national average in children attending schools in most deprived deciles [18]. In this study we demonstrate inequalities (based on child’s area of residence) in thinness in the NCMP using international cut-offs, and differentiating between mild and moderate/severe thinness. We supplement these findings with analyses from a nationally representative cohort collected over a similar period, which indicate that these inequalities exist when measuring disadvantage at the individual level and while controlling for early life characteristics.

The prevalence of thinness in income rich countries has been shown to be higher in certain ethnic minority groups, such as Asian and Middle Eastern children [29–32]. This has prompted suggestions that the IOTF BMI cut-offs may overestimate thinness in these groups [32], although others have suggested that these elevated levels may be due to greater levels of disadvantage experienced by immigrant populations [31]. In the MCS, the prevalence of thinness was substantially higher in South Asian and ‘Other’ ethnic groups when compared to White children; and in the UK these groups tend to live in more disadvantaged circumstances [33]. In the absence of conclusive evidence to indicate whether the higher rates of thinness in South Asian groups are a result of inter-generational exposure to poverty, or biological differences, we repeated our analyses in White, Black and Mixed ethnicity children only, to remove the potential confounding effects of South Asian ethnicity. The elevated (adjusted) relative risks of thinness in children in the most disadvantaged socio-economic categories were then attenuated but remained statistically significant in almost all socio-economic measures.

Socio-economic inequalities in childhood thinness might be set early in life. For example, compared to their more advantaged peers, children from disadvantaged backgrounds are more likely to be born prematurely or with a low birthweight, their mothers are more likely to smoke in pregnancy, and they are less likely to have been breastfed [34]. The Scottish study reported earlier found that area level inequalities in thinness remained after adjustment for birthweight [17], however to our knowledge no study has accounted for other important early life characteristics. We found that individual-level inequalities in thinness were unchanged after adjustment for birthweight as well as maternal age, health behaviours in pregnancy, parity, gestational age and breastfeeding, indicating that later factors are likely to be important.

### Strengths and limitations

The MCS is a large and contemporary cohort, and when sample weights are applied, analyses should be generalisable to the UK population. However our analyses were limited to 88 % of children with BMI data at a minimum of one time point; and we cannot rule out bias due to attrition. The NCMP aims to collect the heights and weights of all 4–5 (and 10–11) year old children attending state maintained schools in England [18], which make up around 95 % of all children. Approximately 4 % of 4–5 year olds were attending independent primary schools in 2012 [35]. It is likely that children attending independent schools are more from advantaged backgrounds, because, unlike state schools, they charge fees (in 2013 the average independent junior school fee was in excess of £10,000 per annum [36]). Through including IMD in our models we will have accounted for some of the socio-economic bias, although we acknowledge that certain groups will remain under-represented in the study. Our results are also susceptible to response bias; between 89 % (in 2007/8) and 94 % (in 2011/12) of eligible children took part in the NCMP, and it is possible that overweight or thin children would be less likely to be included. Finally, while the NCMP and MCS are intended to be nationally representative (of England and the UK respectively), both are likely to under-represent very disadvantaged children. Therefore it is possible that our analyses have underestimated the prevalence of thinness, and the extent of the relative difference between the most and least advantaged groups.

Despite the MCS being the largest cohort currently available in the UK, statistical power was limited when estimates of thinness were broken down by SECs. Therefore, we pooled data collected at ages three, five and seven years, controlling for sweep and adjusting standard errors to account for repeated measures. Still, confidence intervals were wide, and it was not possible to examine inequalities in more severe levels of thinness or in ethnic minority populations. It was not possible to fully account for the survey design in the MCS as well as the repeated measures at each sweep. However models were run for each sweep individually, adjusting for sample and response weights, and socio-economic patterns were similar (albeit with some loss of power).

BMI cut-offs provide a good measure of adiposity for monitoring weight at the population level; however they cannot provide an accurate measure of fatness (or lean mass) in individuals. Cole’s international cut-offs were created using nationally representative data on almost 200,000 individuals from six high-middle income countries (UK, USA, Brazil, Hong Kong, the Netherlands, and Singapore) [5]. While they provide a practical measure for estimating the burden of thinness for public health purposes, a major assumption is that the cut-offs have the same meaning across countries. We found that MCS children from South Asian and ‘Other’ ethnic groups had higher rates of thinness; the social gradient in thinness was attenuated but remained when these children were excluded from our analyses. Children of mixed ethnicity were retained in our analyses and some of these were White-Asian, however they made up a very small proportion of the sample (around 1 %) and sensitivity analyses indicated that inclusion of this group did not confound our results. There were insufficient numbers to examine socio-economic inequalities in thinness for individual ethnic groups despite the oversampling of children living in areas with high proportions of ethnic minority groups in the MCS. Alternative studies, such as the Born in Bradford cohort, may be better placed to examine this. Further research is also required to validate thinness cut-offs in different ethnicities, and to examine whether the consequences of thinness (as defined with these cut-offs) are the same across ethnicities.

It was only possible to examine socio-economic inequalities in the NCMP using an area level measure of deprivation, which was captured using the 2010 classification and cannot account for area level changes in disadvantage that may have occurred across the period examined. Similarities between the area- and individual-level analyses in the MCS indicate that the gradient will also exist at the individual level, although there is a possibility that these patterns might be attenuated after accounting for individual ethnicity (which we were not able to do in the NCMP). Nevertheless, that thinness is higher in more deprived areas is an important finding, which may be used for planning local policy and practice. We examined several different measures of SECs in the MCS; however we acknowledge that it is impossible capture the complexities of socio-economic position in its entirety, nor the experiences of individual families from different socio-economic groups. The consistent patterns seen according to several of the SECs measures and in two different datasets indicate that thinness may be an issue related to disadvantage, although we have not been able to explore this further. Future research should investigate potential explanations for our findings, which might include serious health conditions that lead to weight loss and are more prevalent in disadvantaged groups, or food poverty. A study of nutrition and poverty in the UK reported a higher consumption of unhealthy food items and lower intake of essential vitamins and minerals among the poorer groups [37]; and there is evidence to suggest that inequalities in diet and access to food have widened since the recession [4]. However, our findings indicate the socio-economic inequalities in thinness were present prior to the recession.

In the MCS we were able to examine the potential confounding role of a number of early life characteristics, including birthweight, breastfeeding and maternal pre-pregnancy BMI. These were all based on maternal report and are therefore susceptible to recall and report bias. A systematic review of studies comparing self-reported and objectively measured BMI in adults found that self-reported BMI tends to be under-estimated, and that the degree of individual variation is large [38]. Therefore it is possible that the prevalence of thinness in MCS mothers was overestimated. A systematic review [39] found that maternal reports of breastfeeding duration are reliable and valid when the period of recall was less than three years (as in the MCS). A comparison of maternal reported birthweights and birth registry data was carried out for 84 % of the MCS babies who were born in England and Wales. 92 % of women reported birthweight within 100 g if the registry record, although there was some variation by ethnicity. For example just 63 % of Bangladeshi mothers report birthweight within 100 g; however mothers from all ethnic groups were as likely to overestimate as underestimate birthweight, and therefore our adjusted results should suffer from limited bias in this respect [40]. Mothers with lighter babies tended to overestimate birthweight, although mean differences were very small, and so the underestimation of low birthweight should be limited.

It has been postulated that thinness in children may arise due to inter-generational transference from an underweight parent [29]. We found that underweight mothers in the MCS were more likely to have a thin child, but that this did not explain the social gradient in childhood thinness (nor did adjustment for birthweight). Future research should examine the extent to which the inter-generational transference of thinness is due to genes or shared environments.

In this paper we have focussed on relative differences in weight status. Absolute differences in the prevalence of thinness were relatively small (particularly in the NCMP). However, in light of the current period of austerity, we believe these patterns warrant further scrutiny.

## Conclusions

Thinness has been linked to reduced health and life expectancy, and our study has shown that around 5–6 % of young children are thin. Furthermore, children from the most deprived groups are at elevated relative risk of thinness. The coexistence of childhood thinness and obesity in more disadvantaged groups implies that there may be common environmental and social risk factors. Researchers and policymakers should therefore focus on the environmental determinants of thinness as well as obesity. A better understanding of how weight interventions and wider public health messages (which tend to focus more on overweight and obesity) might influence thinness at the population level is required [41]. A survey of middle-grade doctors working in hospitals identified low levels of knowledge around the identification and potential health risks of severe thinness in children [42], and the authors concluded that better training for paediatric doctors is required. Due to the negative health consequences of thinness, increasing awareness and support amongst primary care health professionals might also be of benefit.

Our analyses indicate that it is not early life characteristics, such as birthweight, that are causing socio-economic inequalities in thinness. It is therefore likely that factors later in childhood, such as diet and exercise, may be important. It is essential to continue to monitor the burden and social distribution of thinness into the future, since recent rises in food prices and the cost of living may exacerbate the patterns observed in our study. Policies designed to buffer families against adverse economic trends and their consequences may help to tackle inequalities in thinness, but regardless will be beneficial for child wellbeing more generally.

## Additional file

Additional file 1:
**Weighted % (n) thin, overweight or obese at three, five and seven years, overall and by demographic and early life characteristics.** (DOCX 29 kb)

## Abbreviations

BMI: Body mass index
CI: Confidence interval
IMD: Index of Multiple Deprivation
IOTF: International Obesity Task Force
MCS: Millennium Cohort Study
NCMP: National Child Measurement Programme
NS-SEC: National Statistical Socio-economic Classification
n: Number
RRR: Relative risk ratio
SEC: Socio-economic circumstances
SOA: Super Output Area
WHO: World Health Organisation
