# Supplementary material for: Disadvantaged children at greater relative risk of thinness (as well as obesity): a secondary data analysis of the England National Child Measurement Programme and the UK Millennium Cohort Study
Source: Int J Equity Health. 2015 Aug 5;14:61. doi: 10.1186/s12939-015-0187-6 (PMC4524014; doi:10.1186/s12939-015-0187-6)
Supplement: Additional file 1: — Weighted % (n) thin, overweight or obese at three, five and seven years, overall and by demographic and early life characteristics. (DOCX 29 kb) [file 12939_2015_187_MOESM1_ESM.docx]

|  | | **Three years (n=15,381)** | | | | | | | **Five years (n=15,041)** | | | | | **Seven years (n=13,681)** | | | | | |
| --- | --- | --- | --- | --- | --- | --- | --- | --- | --- | --- | --- | --- | --- | --- | --- | --- | --- | --- | --- |
|  | | **Thin** | **Over-weight** | | **Obese** | **P-value** | | | **Thin** | **Over-weight** | | **Obese** | **P-value** | **Thin** | **Over-weight** | **Obese** | | **P-value** | |
| ***Demographic factors*** | | | | | | | | | | | | | | | | | | | |
| **Sex** | Male | 4.25 (315) | 17.69 (1,191) | | 4.66 (337) | 0.0976 | | | 4.61 (375) | 13.62 (1,011) | | 5.28 (399) | <0.001 | 5.59 (385) | 12.75 (829) | 4.91 (347) | | <0.001 | |
|  | Female | 4.85 (333) | 18.12 (1,241) | | 5.41 (365) |  |  |  | 3.79 (297) | 17.37 (1,252) | | 5.87 (431) |  | 6.01 (385) | 16.27 (1,058) | 6.46 (426) | |  |  |
| **Ethnicity** | White British/ White other | 3.51 (396) | 18.61 (2,221) | | 4.69 (578) | <0.001 | | | 3.33 (412) | 15.68 (2,009) | | 5.14 (663) | <0.001 | 4.87 (528) | 14.29 (1,631) | 5.07 (605) | | <0.001 | |
|  | South Asian | 16.58 (209) | 9.8 (137) | | 6.05 (72) |  |  |  | 13.65 (198) | 11.22 (142) | | 7.19 (91) |  | 17.15 (203) | 14.06 (154) | 8 (93) | |  |  |
|  | Black/ Black British | 4.46 (21) | 15.76 (65) | | 12.72 (49) |  |  |  | 4.81 (26) | 21.03 (99) | | 12.57 (56) |  | 5.54 (18) | 20.59 (89) | 13.39 (54) | |  |  |
|  | Mixed | 7.31 (34) | 17.31 (72) | | 4.84 (21) |  |  |  | 7.43 (36) | 14.8 (64) | | 7.44 (31) |  | 6.89 (29) | 16.69 (54) | 7.89 (30) | |  |  |
|  | Other (incl. Chinese) | 14.15 (28) | 15 (24) | | 6.38 (12) |  |  |  | 11.54 (27) | 7.98 (17) | | 4.97 (11) |  | 13.67 (25) | 11.81 (22) | 4.95 (10) | |  | |
| ***Early life factors*** | | | | | | | | | | | | | | | | | | | |
| **Birth weight** | Low <2.5kg | 12.54 (115) | 9.23 (73) | 4.54 (33) | | | <0.001 | 11.14 (119) | | | 9.43 (81) | 4.44 (43) | <0.001 | 13.34 (107) | 11.76 (91) | | 5.4 (44) | | <0.001 |
|  | Normal >=2.5kg <=4.5kg | 4.1 (528) | 18.18 (2,282) | 5 (646) | | |  | 3.83 (552) | | | 15.68 (2,106) | 5.56 (761) |  | 5.39 (658) | 14.59 (1,738) | | 5.67 (711) | |  |
|  | High 4.5kg+ | 1.56 (4) | 32.32 (69) | 8.16 (19) | | |  | 0.12 (1) | | | 26.46 (70) | 8.6 (21) |  | 1.42 (3) | 20.35 (711) | | 6.77 (16) | |  |
| **Mother’s weight pre- pregnancy** | Underweight <18.5BMI | 8.58 (56) | 12.22 (86) | 1.94 (13) | | | <0.001 | 9.08 (77) | | | 9.76 (76) | 2.59 (21) | <0.001 | 10.9 (80) | 12.44 (72) | | 2.25 (15) | | <0.001 |
|  | Normal | 4.71 (411) | 16.58 (1,369) | 3.53 (317) | | |  | 4.54 (440) | | | 13.52 (1,223) | 3.77 (349) |  | 6.33 (516) | 12.05 (976) | | 3.67 (307) | |  |
|  | Overweight | 3.42 (95) | 21.11 (547) | 7.6 (192) | | |  | 2.76 (81) | | | 19.71 (544) | 8.2 (220) |  | 3.94 (97) | 18.61 (462) | | 8.44 (213) | |  |
|  | Obese | 1.86 (26) | 25.09 (215) | 8.74 (67) | | |  | 1.49 (16) | | | 23.48 (203) | 12.86 (109) |  | 1.77 (12) | 24.03 (179) | | 13.63 (111) | |  |
|  | Morbidly obese | 2.81 (10) | 23.31 (76) | 15.77 (50) | | |  | 1.4 (5) | | | 26.74 (87) | 15.97 (61) |  | 1.8 (6) | 26.75 (78) | | 17.61 (58) | |  |
| **No. live births** | Mean (SE) | 1.91 (0.056) | 1.89 (0.024) | 1.99 (0.043) | | |  | 1.92 (0.054) | | | 1.91 (0.029) | 1.97 (0.038) |  | 1.92 (0.046) | 1.88 (0.028) | | 2.03 (0.041) | |  |
| **Gestational age** | Pre-term (<37 wks) | 9.78 (64) | 13.16 (80) | 5.48 (35) | | | <0.001 | 8.7 (61) | | | 11.53 (78) | 5.31 (34) | <0.001 | 10.26 (55) | 13.67 (77) | | 4.35 (29) | | 0.0003 |
|  | Early/full-term (37-41 wks) | 4.4 (467) | 17.67 (1,767) | 4.89 (503) | | |  | 4.18 (493) | | | 15.05 (1,631) | 5.44 (598) |  | 5.79 (571) | 14.09 (1,353) | | 5.83 (579) | |  |
|  | Past-term (>41 wks) | 3.97 (112) | 20.13 (571) | 5.27 (151) | | |  | 3.04 (103) | | | 18.1 (535) | 5.76 (181) |  | 4.87 (137) | 16.07 (436) | | 5.34 (154) | |  |
| **Breastfeeding** | None | 4.67 (198) | 19.67 (850) | 5.76 (243) | | | 0.0008 | 4.32 (192) | | | 16.72 (802) | 6.29 (296) | 0.0002 | 5.61 (210) | 15.4 (640) | | 6.75 (281) | | 0.0007 |
|  | Less than 4 months | 4.68 (246) | 17.6 (873) | 5.22 (262) | | |  | 4.19 (262) | | | 15.4 (820) | 6.13 (323) |  | 5.4 (279) | 14.93 (701) | | 5.7 (289) | |  |
|  | 4 months or more | 4.31 (204) | 16.67 (705) | 4.13 (194) | | |  | 4.14 (218) | | | 14.42 (637) | 4.25 (208) |  | 6.43 (279) | 13.09 (289) | | 4.59 (203) | |  |
| **Smoking in pregnancy** | Non-smoker before pregnancy | 4.84 (473) | 17.38 (1,547) | 4.74 (428) | | | 0.1441 | 4.47 (487) | | | 15.08 (1,415) | 4.95 (485) | 0.0037 | 6.32 (561) | 13.98 (1,185) | | 5.02 (456) | | 0.0007 |
|  | Stopped when pregnant | 4.45 (69) | 17.97 (291) | 4.32 (75) | | |  | 4.26 (71) | | | 14.32 (271) | 5.79 (107) |  | 5.73 (92) | 14.88 (255) | | 5.7 (99) | |  |
|  | Yes- Smoked <1-5/day | 3.48 (47) | 18.82 (265) | 6.44 (90) | | |  | 3.83 (59) | | | 16.99 (259) | 6.52 (92) |  | 5.59 (67) | 15.24 (206) | | 6.54 (85) | |  |
|  | Yes- Smoked 6-10/day | 4.27 (38) | 19.72 (177) | 5.43 (57) | | |  | 2.91 (31) | | | 17.51 (177) | 7.9 (76) |  | 3.78 (26) | 15.49 (128) | | 8.56 (75) | |  |
|  | Yes- Smoked 11+/day | 3.78 (20) | 19.93 (141) | 6.79 (46) | | |  | 3.11 (22) | | | 17.09 (126) | 7.64 (65) |  | 3.08 (22) | 17.22 (104) | | 7.89 (55) | |  |
| **Drinking in pregnancy** | Never | 5.13 (512) | 18.39 (1,715) | 5.22 (514) | | | 0.0010 | 4.65 (531) | | | 15.89 (1,598) | 6.07 (631) | 0.0008 | 6.29 (586) | 14.95 (1,343) | | 6.4 (602) | | <0.001 |
|  | Light | 3.49 (107) | 16.88 (537) | 4.46 (134) | | |  | 3.33 (110) | | | 14.58 (497) | 4.31 (143) |  | 4.98 (146) | 13.32 (417) | | 4.04 (128) | |  |
|  | Moderate | 3.13 (15) | 18.46 (102) | 4.16 (26) | | |  | 3.38 (19) | | | 14.76 (98) | 3.59 (24) |  | 3.53 (18) | 11.9 (63) | | 3.83 (23) | |  |
|  | Heavy/Binge | 2.7 (14) | 15.14 (72) | 6.62 (25) | | |  | 3.34 (12) | | | 14.44 (62) | 7.21 (28) |  | 4.55 (17) | 17.08 (58) | | 5.66 (20) | |  |

**Additional file 1. Weighted % (n) thin, overweight or obese at three, five and seven years, overall and by demographic and early life characteristics**

Missing data: age (0), BMI (1001), smoking in pregnancy (58), Alcohol in pregnancy (28), gestational age (155), birthweight (30), breast feeding (14), number of live births (496).
